# Recent Progress in MOF-Based Electrochemical Sensors for Non-Enzymatic Glucose Detection

**DOI:** 10.3390/molecules28134891

**Published:** 2023-06-21

**Authors:** Ziteng Li, Wen Zeng, Yanqiong Li

**Affiliations:** 1 College of Materials Science and Engineering, Chongqing University, Chongqing 400030, China; 202109021142t@cqu.edu.cn; 2 School of Electronic Information & Electrical Engineering, Chongqing University of Arts and Sciences, Chongqing 400030, China; 20170031@cqwu.edu.cn

**Keywords:** MOF, glucose, enzyme-free, electrochemical sensing

## Abstract

In recent years, substantial advancements have been made in the development of enzyme-free glucose sensors utilizing pristine metal-organic frameworks (MOFs) and their combinations. This paper provides a comprehensive exploration of various MOF-based glucose sensors, encompassing monometallic MOF sensors as well as multi-metal MOF combinations. These approaches demonstrate improved glucose detection capabilities, facilitated by the augmented surface area and availability of active sites within the MOF structures. Furthermore, the paper delves into the application of MOF complexes and derivatives in enzyme-free glucose sensing. Derivatives incorporating carbon or metal components, such as carbon cloth synthesis, rGO-MOF composites, and core–shell structures incorporating noble metals, exhibit enhanced electrochemical performance. Additionally, the integration of MOFs with foams or biomolecules, such as porphyrins, enhances the electrocatalytic properties for glucose detection. Finally, this paper concludes with an outlook on the future development prospects of enzyme-free glucose MOF sensors.

## 1. Introduction

Glucose, as the primary source of energy for cells, plays a crucial role as an essential organic compound in human blood [1]. However, diabetes mellitus, characterized by abnormally high glucose levels in the bloodstream, has become a prevalent chronic disease in modern society [2,3,4]. According to the International Diabetes Federation (IDF), the global count of individuals aged 18–99 years with diabetes reached 451 million in 2017, with a projected increase to 693 million by 2045. Alarmingly, nearly half of these cases (49.7%) remain undiagnosed [2]. Prolonged hyperglycemia poses significant risks to various organs, resulting in severe complications such as coronary heart disease, renal failure, and cardiovascular disorders. These complications have a substantial impact on the overall well-being and life expectancy of affected individuals [3,4]. Consequently, the timely, rapid, and accurate detection of blood glucose levels in clinical settings assumes paramount importance [5,6].

So far, researchers have developed various methods for measuring glucose concentration, such as optical, conductometric in [7,8], and electrochemical biosensor methods [7,9,10]. Among them, the operation of the colorimetric detection method is characterized by its complexity and lack of simplicity, and it is susceptible to significant errors. Similarly, the detection process associated with the fluorescence analysis method is intricate, and it requires expensive equipment. Despite the chemiluminescence method’s advantages of high detection sensitivity and rapid speed, it suffers from drawbacks including the consumption of luminescent material during the testing process and the inherent instability of luminescence, ultimately leading to inconsistent detection results. The electrochemical biosensor approach has garnered considerable attention as a potential detection technique due to its notable attributes, including high sensitivity, rapid reaction time, a wide linear range, and straightforward operation. A typical electrochemical sensor comprises three essential components: the identification element, the transducer, and the signal output device. In the case of electrochemical glucose sensors, the recognition element typically consists of a catalytic material that is sensitive to glucose and immobilized on the electrode. This type of sensor can be categorized into two main groups based on the classification of the modified substance: enzyme-based sensors and enzyme-free sensors. However, among these categories, the activity of glucose oxidase, an enzyme-based sensor, can be easily influenced by environmental factors such as temperature, humidity, and pH value, primarily due to inherent deficiencies in the enzyme itself [11].

Moreover, the production cost of the enzyme is high, and the enzyme cannot be reused after inactivation, so it is challenging to meet the increasing demand of people. The electrode material used in an enzyme-free sensor facilitates glucose oxidation directly, successfully addressing the challenge of enzyme activity loss during utilization. The sensor has the advantages of a simple preparation process, low cost, high sensitivity, and excellent development potential. The catalytic effect of most electrocatalysts for non-enzymatic glucose sensors is related to the metal centers of the materials. Among them, metal-organic framework compounds (MOFs) [12,13,14,15,16], which have excellent properties, such as a large surface area, regular and customizable pores, and excellent microscopic morphology, are extremely porous materials made of metal ions and organic ligands through coordinate covalent bonds. In the past two decades, these novel functional materials have garnered the attention of many researchers and contributed to the advancement of MOFs-based materials (including raw MOFs, MOFs composites, and MOFs derivatives, etc.) widely used in energy storage and conversion, catalysts, gas storage, electrochemical sensing, and other fields [17,18,19,20,21,22]. The characteristics of MOF materials offer significant advantages for their utilization in electrochemical sensing applications: (1) MOFs possess a highly porous structure with adjustable porosity, which allows for the efficient diffusion and adsorption of target analytes. The presence of unsaturated metal coordination sites provides active sites for specific interactions with the analyte molecules. (2) MOFs exhibit a large specific surface area, which provides ample space for analyte adsorption and enhances the contact between the analyte and the electrode surface. This increased surface area facilitates efficient mass transfer, leading to improved signal response and enhanced detection sensitivity. (3) The remarkable catalytic capabilities of MOFs render them highly suitable as active materials for catalytic electrodes, owing to their exceptional catalytic performance. (4) Pores and hollow structures of specific sizes may exclude interfering substances utilizing the size exclusion effect, thereby improving the interference immunity of the prepared samples. Therefore, MOF-based materials can also fabricate active electrode materials for enzyme-free sensors. 

However, pure MOFs have poor chemical and electronic conductivity, low mechanical stability, and poor electrocatalytic ability. These shortcomings greatly limit the electrochemical performance of enzyme-free glucose sensing. In addressing these challenges, researchers typically approach the issue from the following perspectives [23,24,25]. First, the combination of conductive materials with MOFs has emerged as a prominent strategy to fabricate composite materials with enhanced conductivity and durability. Notable examples of such conductive materials encompass carbon materials, metal/metal oxide nanoparticles, conductive polymers, and graphene materials [26,27,28]. Particularly, graphene aerogels (GAs) have gained significant attention in recent years as a novel and versatile porous material widely explored by researchers [29,30,31]. GAs exhibit a high porosity, substantial surface area, tunable electrical conductivity, and low density. The integration of GAs with MOFs provides a greater number of active sites and facilitates the preservation of the intrinsic characteristics of both components, thereby giving rise to a myriad of intriguing and synergistic effects. Second, the incorporation of other materials possessing excellent conductivity into MOFs results in a synergistic effect, leading to a notable enhancement in the overall conductivity of the active material [32,33]. Noteworthy examples of such materials include nanoparticles, conductive polymers, nanofibers, biomolecules, and metal-organic frameworks. By combining these components, composite materials are formed, effectively broadening their applications in the realm of electrochemistry. Third, the combination of one MOF with another, or the integration of multiple MOFs, has emerged as a novel and effective approach for functionalizing MOFs. By combining two MOFs, core–shell MOF@MOF [34,35] compounds can be created while retaining the structural, chemical, and physical benefits of the two distinct MOFs. At the same time, the MOF@MOF structure can also produce some unexpected novel synergistic effects. Furthermore, non-enzymatic glucose sensors frequently employ precious metals, such as Pt, as electrodes. However, precious metals are susceptible to poisoning when exposed to elevated concentrations of Cl^-^. This susceptibility significantly impacts the stability, detection, and durability of the materials. Consequently, it is recommended to incorporate Cl^-^ during the assessment of interfering agents or to consider alternatives to precious metal electrodes. MOF materials exhibit high responsiveness to the oxidation of biomolecules on the electrode. However, interference from easily oxidizable substances can be mitigated by adjusting the working potential within an optimal range (−0.2 V–0 V, relative to the Ag/AgCl reference electrode). Moreover, different MOFs display selective permeability towards distinct biomolecules. This selectivity can be enhanced by designing the pore size of MOFs or modifying their surface charge, effectively preventing the contact between interfering components and the electrode. These approaches contribute to improved selectivity of the material towards glucose.

In this comprehensive review, we aim to systematically summarize the diverse applications of metal-organic frameworks (MOFs) in glucose sensing. We provide an overview of the synthesis strategies and properties of various MOFs utilized in this field. Additionally, we compare the advantages and disadvantages of different non-enzymatic glucose sensors that are based on MOFs. Finally, we delve into the challenges and opportunities that exist within this area of research.

## 2. Pristine MOF and MOF Conjugates as Non-Enzymatic Glucose Sensors

Excellent enzyme-free glucose sensors can be fabricated using a single MOF or by synergistically combining two or more MOFs. In the majority of instances, the amalgamation of multiple MOFs leads to a substantial enhancement in the material’s sensitivity towards glucose. In the first section of this chapter, we introduce a non-enzymatic glucose sensor based on a metal-based MOF composition. In the second section, several MOF-based glucose sensors are introduced.

### 2.1. Enzyme-Free Glucose Biosensor Based on Monometallic MOF

For example, CuO polyhedrons-MOF grow in three-dimensional flexible carbon cloth [36]. The CC(carbon cloth) substrate has more area than conventional two-dimensional substrates, excellent mechanical strength, high electrical conductivity, and good corrosion resistance. Measurements were made in alkaline solution using three electrodes, with the working electrode being a carbon cloth. The material showed a high response of 13,575 μA mM^−1^ cm^−2^ at an operating voltage of 0.55 V. The detection limit was 0.46 μM. Moreover, the substrate does not need conductive adhesives. The CuO product preserves the porous structure of the MOF. The sensor was highly selective for glucose against 1 mM glucose and 0.1 mM uric acid (UA), ascorbic acid (AA), dopamine (DA), sucrose, NaCl, and KCl. Furthermore, the Ni_3_(2,3,6,7,10,11-hexaiminotriphenylene)_2_MOF (Ni_3_(HITP)_2_MOF) was prepared by adding triethylamine to the MOF preparation solution [37]. Triethylamine can improve the material’s conductivity, thereby improving the catalytic oxidation ability of Ni-MOF for glucose. The Ni-MOF has a linear relationship with 0–10 MM glucose and has good anti-interference. Working electrodes, counter electrodes and reference electrodes were selected, respectively: glass carbon electrode (GCE), platinum rod, and saturated calomel electrode (SCE). However, the stability test was only tested for seven days, and the reference value could have been better. Gumilar et al. [38] prepared benzene dicarboxylic acid (BDC)-based MOFs composed of two-dimensional nanosheets using an oil bath method. A three-electrode system was used in the electrochemical experiment. MOF suspensions were prepared in isopropanol solution. N_2_ adsorption–desorption experiments showed that the specific surface area of the four MOFs was Zr-BDC > Mn-BDC > Cu-BDC > Ni-BDC. These data demonstrate a smaller surface area compared to previously reported two-dimensional MOFs, potentially due to the blockage of internal channels by PVP (polyvinyl pyrrolidone). The redox peaks of the four MOFs were measured by the electrochemical method, and the current density of the four materials was higher than that of the bare electrode. In addition, the Ni-BDC redox peak was the most pronounced. Further tests showed that the current density of the flaky Ni-BDC was more significant than that of the bulk Ni-BDC, which may be due to the large specific surface area providing more reaction sites, and the cross structure providing diffusion paths for glucose molecules. The anti-interference property of the material was also tested. The material also had a specific current density for uric acid, but the value was much smaller than that observed for glucose. Other molecules, such as ascorbic acid and maltose, do not cause a reaction.

A Ni-MOF, prepared by using nickel salt and HHTP(2,3,6,7,10,11-hexahydroxytriphenylene), was used as a non-enzymatic glucose sensor [39]. The electron transport was ensured by the delocalized π bond in the ligand. The researchers tested the current response of Ni-MOF to glucose at different potentials, with 0.55 V being the optimal working potential. The material demonstrated a detection range of 0.001–8 mM, with a limit of detection of 0.66 M (S/N = 3). It exhibited a rapid response and a high sensitivity of 21,744 μA mM^−1^ cm^−2^ (3 s). The sensor displayed excellent selectivity, reliability, and repeatability, making it suitable for monitoring glucose in actual samples. Furthermore, the ultrasonic treatment at different times facilitated the longitudinal expansion of the layered material. The researchers hydrothermally prepared sheet-packed Ni-MOFs, which were subsequently sonicated for varying durations (0, 30, 60, and 120 min) [40]. The XRD results revealed an increase in the intensities of the (010) and (020) crystal planes of Ni-MOF, while the intensities of the (100), (200), and (10-1) crystal planes decreased with increasing ultrasonic time. However, when the ultrasonic time reached 120 min, the strength of all crystal planes decreased, possibly due to the pulverization of the layered materials, as confirmed by SEM analysis. Additionally, ultrasonic treatment aided in the dissolution of the remaining Na^+^ ions in the interlayer into the solvent. During the electrochemical activation of the MOF, Ni^2+^ was oxidized to Ni^3+^ and Ni^4+^, as ultrasonication for 60 min can enhance both oxidation modes simultaneously. The active surface area of Ni-MOF60 was much larger than the electrode surface area after analyzing the Nyquist diagram, which proved the influence of ultrasonic treatment on the material properties. The lowest detection limit of the material was 8.97 μM, which was higher than other MOF non-enzymatic glucose sensors. To assess the selectivity of the material, biomolecules and NaCl were introduced into an alkaline solution, and the resulting impact on current density was evaluated. Remarkably, even after a 60 min test, the current density only experienced a marginal decrease of 10%. This outcome highlights the material’s robust selectivity, as it exhibited minimal interference from the introduced biomolecules and NaCl. Furthermore, the experimental team went on to develop a sweat glucose detection sensor. The device consists of a solid electrolyte, disposable electrodes, a counter electrode, and a reference electrode. Sweat can be tested for glucose concentration at 20 mV s^−1^. This innovative sensor design proved to be highly effective during a one-day sweat test conducted with volunteers, showcasing its exceptional practicality and reliability in real-world scenarios. The main performance parameters of a glucose sensor based on monometallic MOF are shown in Table 1.

Another CuO nanorod fabricated using Cu_3_(benzene-1,3,5-tricarboxylate)_2_ (Cu_3_(BTC)_2_) was also shown to yield excellent performance on glucose [42]. The difference was that the material was synthesized using a solvothermal method. The response to glucose was as high as 1523 μA mM^−1^ cm^−2^ with a minimum detection limit of 1 μM. The material proved to be highly stable. When the material was tested on an actual human serum sample, the results showed that the material had a highly selective response to glucose.

**Figure 1:**
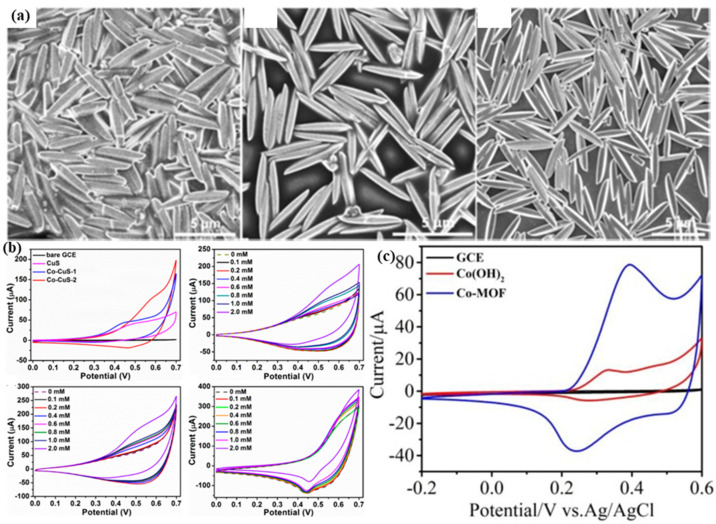
(**a**) SEM images of Cu-MOF(1–3) [53]. (**b**) CV curves of materials in 0.1 M NaOH [53]. (**c**) CV of materials in 0.01 M NaOH containing 1 mM glucose [47].

**Figure 2:**
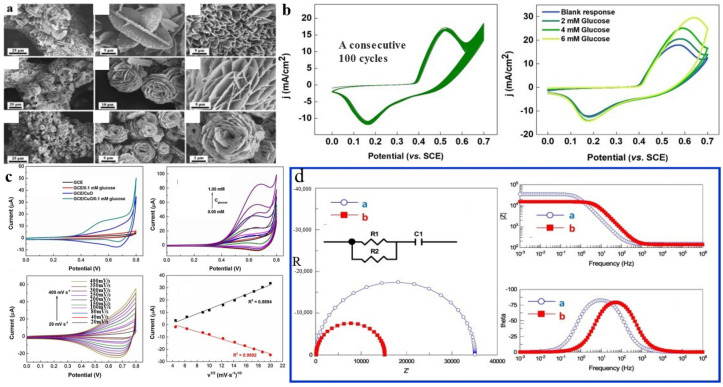
(**a**) SEM images of Cu_X_Co_X_-MOF/NF [54]. (**b**) CV curves of materials [48]. (**c**) CVs obtained for different glucose [49]. (**d**) Nyquist and Bode amplitude of bare GC and GC/CuO electrodes [50].

Zeraati et al. [44] successfully synthesized Ni-MOFs at a significantly low cost. The MOF had an average particle size of 80 nm. The researchers did not test the material’s resistance to interference, stability, or accuracy against natural materials. The electrode exhibited a detection limit of 0.76 μM and a wide detection range of 1–1600 μM. The CV curves of the Co-MOF designed by Zhang et al. [47] are shown in Figure 1c. An ultra-thin Ni-MOF nanosheet was synthesized by a simple one-pot strategy for glucose detection [48]. The electrodes were prepared via a drop coating method. The electrochemical measurements were carried out in a KOH solution. The redox peak of Ni^2+^ and Ni^3+^ interconversion appeared at 0.49 V. Ni^3+^ can reduce glucose to gluconolactone in the presence of hydroxyl ions. The pH of the working solution was set at pH = 13. The optimum working potential was 0.55 V. The current density also increased significantly with the continuous addition of glucose. The detection limit of Ni-MOFNs/GCE was 0.6 μM. The CV curve shown in Figure 2b. The researchers conducted an interference test on the material by selecting a limited number of biomolecules, but they did not specifically include the common Cl^-^ as an interference object. The results revealed that the material exhibited minimal current response to the tested biomolecules, indicating that their presence did not significantly affect glucose detection. However, it should be noted that the absence of testing with chloride ions may limit the comprehensive assessment of the material’s susceptibility to all potential interferences. In addition, the current response of the material decreased to 93.1% within two weeks, indicating that the stability was general. Nevertheless, the advantage of this paper lies in the simplicity of material preparation, which eliminates the need for hydrothermal methods. This was the most straightforward MOF enzyme-free glucose sensor. In many studies on interference resistance, the standard Cl^−^ (which can cause Cl poisoning and subsequently affect the sensor performance) was often not selected.

Archana et al. [55] added Ni sites to the octahedral Cu-MOF synthesized using traditional methods. The team initially diluted glucose with varying concentrations of NaCl and subsequently conducted electrochemical tests to assess the material’s susceptibility to chloride poisoning. The results showed that the material does not suffer from Cl^-^ poisoning. An actual sample analysis of the material was conducted, and its response correlated consistently with the Accu-Chek Active glucometer, validating its performance. The current response of the sensor remained at 92.8 % for 56 days, indicating good stability. Furthermore, using MOFs as templates to synthesize Cu-Co sulfides showed an excellent response to glucose, as shown in Figure 1a [53]. The porous spun cone-shaped sensor preserves the morphology of the MOF. The linear range for glucose was 0.001–3.66 mM at a 0.55 V potential. The lowest detection limit was 0.1 μM. The cyclic voltammetry (CV) curves are shown in Figure 1b. The prepared three materials, Co-CuS-2, Co-CuS-1, and CuS, have good stability and selectivity. Co-CuS-2 has the best stability and thus was an ideal glucose detection material. The main performance parameters of a glucose sensor based on multi-metal MOF combination are shown in Table 2.

### 2.2. Enzyme-Free Glucose Sensor Based on Multi-Metal MOF Combination

Combining multiple MOFs not only addresses the limitation of low catalytic activity in a single MOF but also results in a significantly increased specific surface area, thereby providing more active sites for glucose catalysis.

In addition, Chen et al. [59] also prepared Cu/Co bimetallic MOF on carbon cloth. Cu was directly deposited on carbon cloth using magnetron sputtering, and then the carbon cloth was immersed in a cobalt salt solution to prepare Cu-Co-ZIF. Different growth times of bimetallic MOF at room temperature will affect the final structure. Based on the growth pattern analysis, within 1–4 h, as the time increases, the material transitions from nanorods to nanosheets gradually. A further lengthening of the reaction time will allow the material to grow into a nanofilm. At the same time, different temperatures (15, 35, and 65 °C) will affect the growth rate of the material. Among them, the active sites of nanosheets were higher than those of nanorods and nanofilms. Therefore, the nanosheet Cu-Co-ZIFs were selected to detect their response to glucose. Although the material prepared using this method was more expensive, the sensitivity of the material (18,680 μA mM^−1^ cm^−2^) was higher than the above. A Cu-Co bimetallic MOF was successfully prepared on a nickel foam substrate using a one-step hydrothermal method [54]. SEM images are presented in Figure 2a. Cu_1_Co_2_-MOF/NF(nanofilm), Cu_1_Co_1_-MOF/NF, and Cu_2_Co_1_-MOF/NF were prepared by adjusting the Cu salt to Co salt ratio. Monometallic MOFs (Cu-MOF and Co-MOF) were also prepared for comparison. The results of SEM showed that the aggregation of Cu_1_Co_1_-MOF/NF and Cu_2_Co_1_-MOF/NF occurred at different degrees, and the aggregation of Cu_2_Co_1_-MOF/NF was the most serious. CV experiments in 0.1 M NaOH solution demonstrated the highest current density for Cu_1_Co_2_-MOF/NF, with a broader redox peak without glucose. This result was attributed to the fact that the redox reaction was more likely to occur when the proportion of Co ions was large. The amperometry detection of glucose demonstrated an optimal working potential of 0.48 V, which was slightly better than most other investigators’ 0.55 V working potential. The detection limit of Cu_1_Co_2_-MOF/NF was 0.023 mM. The sensitivity was 8304.4 μA mM^−1^ cm^−2^. Co and Cu ions play different roles in the experiment. The addition of Co ions enhances sensitivity, while the incorporation of Cu ions expands the linear range. However, excessive amounts of Cu ions can result in structural collapse. The electrochemical surface area (ECSA) calculated by the CV plot also demonstrated that Cu_1_Co_2_-MOF/NF had the most activity checkpoints, resulting in higher sensitivity. The researchers also tested the material’s immunity to interference (the interfering substance contains Cl^−^). The current density was similar to that of glucose alone at similar concentrations of interfering substances and glucose. Nevertheless, no real samples were tested. Nitrogen-doped carbon nanotube MOFs, which were synthesized using Co-Cu-MOFs as precursors, had demonstrated remarkable performance as enzyme-free glucose sensors [68]. The process involved reacting copper nitrate, cobalt nitrate, and cetyltrimethylammonium bromide solutions under magnetic stirring for 1 h to obtain the target product, which was then heated in a tube furnace to dissolve the reaction mixture. After that, the product was dissolved in an aqueous ethanol solution to form an ink solution, which was subsequently coated onto a glassy carbon electrode. The sensor exhibited excellent selectivity and reusability in detecting both H_2_O_2_ and glucose. The CV curve shown in Figure 2c [49].

Ultrathin, NiCo-layered double hydroxide (LDH)/NiCoS array nanostructures were found for Co-MOF using the glucose sensor [69]. This result shows that the 2D hybrid NiCo LDH/NiCoS array nanostructure sensor exhibited excellent electrocatalytic activity towards glucose in a range of 0.001 μM, with a sensitivity of 2167 μA mM^−1^ cm^−2^. CC, as the substrate, reduces the cost of this electrode. The sensor inherits the advantages of 2D nanostructures, with a high specific surface area and more exposed catalytic sites. MOF templates provide a large number of electron transport channels. CC as a substrate offers enhanced biocompatibility and high electrical conductivity. In addition, the ultra-thin structure of the sensor also provides more possibilities for ion exchange. This material also has a good response for H_2_O_2_ detection. The Nyquist and Bode amplitude of the CuO-MOF designed by Arul et al. [50] are shown in Figure 2d. Zou et al. [57] synthesized seven kinds of bimetallic MOFs with different Ni and Co ratios using an ultrasonic method. The color of MOF was related to the ratio of Co, and MOF gradually turned purple with the increase in the Co ratio. The disadvantage was that the researchers used only SEM-EDS and XPS to determine the atomic fraction of the material. N_2_ adsorption–desorption isotherm and Brunauer–Emmett–Teller (BET) results showed that the material’s pore size and specific surface area increased with Co content. The samples with the largest pore size and specific surface area were those of pure Co-MOF. The charge transfer between Ni and Co was proven by theoretical calculation. The conversion of Ni^2+^ to Ni^3+^ and Co^2+^ to Co^4+^ increased as the potential increased, reaching a saturation point at 0.58 V. The adsorption of glucose on the Ni-Co model was calculated using VASP. The findings revealed that glucose was adsorbed onto the unsaturated coordination metal, with the Ni sites exhibiting the highest affinity for adsorption. In the electrochemical test, the material can respond to the lowest glucose of 0.1 μM within 2 s at a low potential of 0.42 V. The amperometry signal was linear for glucose in the range of 0.1 μM–1.4 mM. The lowest detection limit achieved was 0.047 μM, surpassing that of most papers in the field. Interferents selected for the anti-interference test of the material were dopamine (DA), uric acid (UA), ascorbic acid (AA), and paracetamol (AP), which have minimal effects on the current of glucose. However, Cl- was not used as an interferent. The Ni-Co MOF prepared by Li et al. was added with TCPP (Tris chlorisopropyl Phosphate) as a ligand [70]. A different Ni:Co was used to prepare MOFs as in Zou et al.’s [57] work. The working potential was 0.4 V. The detection limit of the sensor was determined to be 0.3 μM (S/N = 3). In the immunity test, the introduction of Cl^-^ did not have a significant impact on the current response. Feng et al. [71] prepared NiCo_2_O_4_ hollow nanocages. When measuring the CV curve, it was found that the anodic peak potential shifted to the right at glucose concentrations greater than 5.0 mM. This phenomenon can be attributed to the higher concentration of glucose compared to hydroxyl groups, resulting in glucose occupying a portion of the hydroxyl sites. The sensitivity of the material was 1306 μA mM^−1^ cm^−2^. The lowest detection limit was 27 mM (S/N = 3), and the highest was 5.1 mM.

Other studies are also worth noting, such as Xue et al. [58] who synthesized Ni@Cu-MOF and Cu-MOF using simple room temperature stirring. The optimal Cu:Ni ratio determined by CV was found to be 3:2. The material exhibited a detection limit of 1.67 μM, a sensitivity of 1703.33 μA mM^−1^ cm^−2^, and a linear range of 5–2500 μM for glucose detection. Furthermore, the material’s anti-interference properties and its ability to detect glucose in human serum were also evaluated. In the study conducted by Kim et al. [63], Cu@Ni MOFs were synthesized using a two-step hydrothermal method. The electrode prepared with the bimetallic MOF demonstrated lower charge transfer resistance compared to the monometallic MOF, indicating that bimetallic materials have the potential to enhance the catalyst’s conductivity and facilitate the catalytic oxidation of glucose. The experiment found that the anodic current increased with the increase in glucose concentration, which indicated that the material had strong catalytic activity for glucose. During the interference test, substances such as dopamine, ascorbic acid, uric acid, hydrogen peroxide, and NaCl were added. The experimental results demonstrate that even when the concentration of the interferent is significantly higher than that of glucose, the observed change in current is minimal. This finding serves as evidence for the excellent anti-interference capability of the sensor. Secondly, the electrode current decreased by 7% after thirty days, indicating that the stability of Cu@Ni MOFs was also excellent. To further establish the reliability of the material, the researcher conducted actual sample detection using human serum samples obtained from a hospital. The results obtained from this analysis exhibited a high level of agreement with the corresponding clinical reports, thus indicating the excellent reliability of the material in real sample testing scenarios.

Li et al. [72] synthesized a new generation of NiO/Cu-TCPP (Tris chlorisopropyl phosphate) hybrid nanosheets that can be used for supercapacitors and glucose sensing. The synthesis method was also relatively simple. Firstly, NiO, Cu(NO_3_)_2_, and PVP were mixed in DMF (dimethyl formamide) and ethanol solution. Subsequently, the solution was transferred into a high-pressure reaction kettle and subjected to heating at 80 °C for a duration of four hours, resulting in the formation of the desired product. The excellent response of the material to glucose was attributed to the catalytic ability of NiO on glucose. The material has a large specific surface area with many pores for electron transport. In the anti-interference experiment, the material had no obvious amperometric response to UA, AA, and KCl solutions alone. However, it produced an obvious amperometric response after glucose was added to the solution, which proved that the material had good anti-interference ability.

Generally, a single MOF may have a limited number of active sites, and it is through the combination of multiple MOFs with different metals that the material’s active site density can be increased, leading to an enhancement in the electrochemical performance for glucose. After that, MOF can be combined with metal or GO to improve the conductivity and electrochemical performance of the material.

## 3. Enzyme-Free Glucose Biosensors Based on MOF Complexes and Derivatives

### 3.1. Enzyme-Free Glucose Sensor Based on MOF with Carbon or Metal

Since the expected effect of glucose response cannot be achieved solely by using MOF alone, carbon can be compounded on the material, such as synthesizing materials on carbon cloth, combining rGO with MOF, or using MOF to wrap noble metals to form a core–shell structure to improve the electrochemical performance of glucose.

The N- and C-doped Ni-MOF prepared through in situ nitridation was utilized as a non-enzymatic glucose sensor [73]. The spherical MOF has high stability, and the surface’s rough structure increases the material’s specific surface area and provides more sites for the glucose reaction. The N1s spectra of XPS showed that there were three different kinds of N ion in the materials: lattice N in Ni_3_N, pyridinic N, and pyrrolic N in nitrogen-doped carbon materials. The sensitivity of Ni_3_N@C was 1511.59 μA mM^−1^ cm^−2^ in the low range (0.001 μM) with a detection limit of 0.3 μM, and 783.75 μA mM^−1^ cm^−2^ in the high range (3–7 mM). Increasing the concentration of glucose will result in the occupation of the active site by some glucose molecules, thereby reducing the sensitivity. The material was tested for anti-interference and stability in bovine serum samples. Ni-MOF with a core–shell structure was synthesized via a hydrothermal reaction, and Ni/NiO@C was obtained by pyrolyzing the resulting material in a tube furnace under a nitrogen (N_2_) atmosphere [74]. The surface of Ni-MOF with a core–shell structure becomes rough after pyrolysis. XPS spectra showed that Ni existed in the material. The concentration of sodium hydroxide solution for the electrochemical test was 0.1 M. The CV curve obtained in the absence of glucose showed a redox peak, which was attributed to the mutual conversion between Ni^2+^ and Ni^3+^ ions. The optimal working potential for the material was determined to be 0.55 V. The current response reached saturation when the glucose concentration exceeded 10 mM. The material’s anti-interference and stability, and glucose detection in human serum samples, were also tested. The results were excellent, indicating that the material has great potential in glucometer research. Hu et al. [75] synthesized a green MOF-74 (Cu) NS (nanosheet) in glass tubes using the in situ growth method. Under alkaline conditions, the material undergoes a conversion from Cu^2+^ to Cu^3+^, and the Cu^3+^ species can facilitate the reduction of glucose, resulting in a glucose-responsive response. The high dependence of the material on the solution pH was found during electrochemical tests. It was found that there was no pronounced redox peak for glucose in the range of pH 9–12, but a redox peak appeared when the pH was greater than 13. To further demonstrate the reliability of the material, the researcher additionally appended common impurities from human serum samples to the test solution to compare the response of the material to glucose with impurities in the solution; overall, there was not much difference. It is worth mentioning that the process for preparing the electrode was in situ growth without calcination. This facilitates the experimental process, leading to a certain degree of cost reduction.

Li et al. [76] successfully obtained Ni-NC_7_H by performing the pyrolysis of hydrothermally-synthesized Ni-ZIF in a H_2_/Ar atmosphere. A comparative experiment was also conducted using Ni-ZIF pyrolyzed under an Ar atmosphere, referred to as Ni-NC-_5_H. High-angle annular dark-field (HAADF) scanning transmission electron microscopy (STEM) images revealed the encapsulation of Ni particles by N and C, significantly enhancing the stability and distribution of active sites in the material and promoting its catalytic activity. CV was employed to investigate the electrochemical reaction of the material. In the absence of glucose, the electrode exhibited a redox peak attributed to the conversion between Ni^2+^ and Ni^3+^. As the glucose concentration increased, the oxidation current also increased. Notably, the catalytic ability of Ni@NC_7_H was considerably higher than that of Ni@NC_5_H. Furthermore, the Nyquist plots of the two materials demonstrated that the charge transfer resistance of Ni@NC_7_H was low, indicating a faster diffusion of electrolyte ions. The researchers also calculated the ECSA of the materials, with Ni@NC_7_H exhibiting the largest ECSA, signifying the presence of a greater number of active sites. The optimal working potential of the material was determined to be 0.6 V, which was higher than that reported by other researchers. The current response speed was less than 1s, and the detection limit was 0.34 μM. The linear response ceased to exist when the glucose concentration exceeded 1.81 mM, likely due to the occupancy of active sites by glucose. Even if the typical biological interference substances and Cl^−^ have little influence on the electrochemical response of the material and the repeatability is excellent, the narrow detection range will affect the application of the material in practice. The researchers used the material to test the electrochemical response of artificial sweat and human serum but did not compare it with clinical reports.

**Figure 3:**
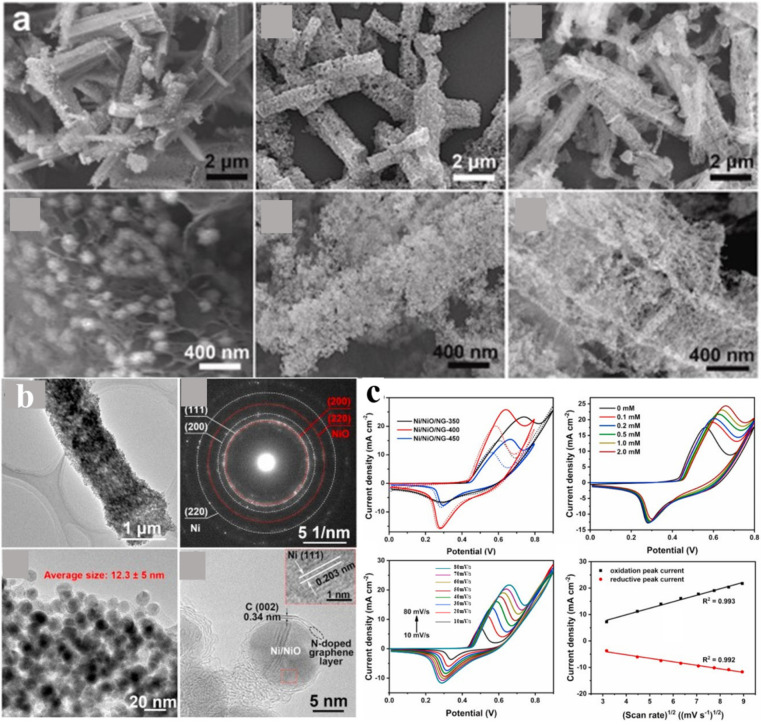
(**a**) SEM images of Ni/NiO/NG [77]. (**b**) TEM and HRTEM analysis of Ni/NiO/NG-400 [77]. (**c**) CV curves of Ni/NiO/NG composites [77].

A novel enzyme-free glucose sensor was fabricated by heating Cu-BTC-MOF [49]. The MOF was converted to porous CuO at high temperatures. The material exhibited a linear response to glucose in the 0.5–2.8 μM with a detection limit below 0.1 μM. In addition, the response to glucose was as high as 934.2 μA mM^−1^ cm^−2^. This material outperformed most metal oxide enzyme-free glucose nanosensors. The excellent performance of the CuO material can be attributed to the following reasons: (1) this CuO material has large pores, which provide good channels for glucose, electron, and ion transport; (2) the abundance of holes on the surface of CuO creates numerous catalytic reaction sites, offering ample opportunities for high response rates. This work obtains a sensor with an excellent performance as a result of annealing Cu-BTC-MOF in air, which provides more ideas and possibilities for future sensor research. Similarly, Wang et al. [77] also synthesized the same sample; however, they employed a different method for MOF synthesis, utilizing the oil bath method, and the heat treatment temperature was only half of that used by Li et al. [76]. The hexagonal prism morphology of the material did not change much due to annealing, Figure 3a. The HRTEM analysis of the material showed that the nanoparticles were encapsulated in a graphite shell, Figure 3b. The CV curves of the materials are presented in Figure 3c. The results of the specific surface area of the samples annealed at different temperatures showed that the samples annealed at 350 °C have the largest specific surface area and the smallest pore size, and the samples annealed at 400 °C have an intermediate specific surface area and average pore size. The XPS spectra of the material treated at 400 °C showed Ni and NiO in the material. An electrochemical analysis showed that Ni/NiO/NG-400 had an enormous peak value, which a multivalent system may cause. The lowest response concentration detected for the material was 1 μM. The detection limit of 0.032 μM was calculated using a linear regression equation of amperometry detection. The amperometry current density of the material was saturated at a 3.568 mM glucose concentration, which was caused by glucose filling the reaction site. Common biomolecules and Cl^−^ were selected for the anti-interference test, which will not have much impact on the detection of glucose by the material. Stability tests over 20 days found a 10% reduction in the current response. The results obtained by utilizing this material for the detection of human serum exhibited no significant difference when compared to the widely recognized phenol-sulfuric acid colorimetric method. Furthermore, the synthesis challenges associated with this work were relatively lower in comparison to the study conducted by Li et al. [76]. The detection of actual samples was more reliable than in the study by Li et al. [76].

Polyhedral NiO/C-MOFs were obtained by subjecting Ni-MOFs to pyrolysis at various temperatures (400, 500, 600, and 800 °C) [78]. After pyrolysis, it was observed that the MOF underwent morphological changes, becoming rough and aggregating into larger particles. At 800 °C, the material completely lost its original morphology. CV testing of Ni-MOF400 in a 0.1 M NaOH solution at a potential of 0.55 V resulted in a linear response range of 5 μM-4.1 mM. The detection limit was determined to be 0.92 μM at a signal-to-noise ratio of 3, with a sensitivity of 2918.2 μA mM^−1^ cm^−2^. The catalytic activity of Ni-MOFs varied depending on the ligands used in their synthesis. Qiao et al. [79] utilized HATP·6HCl as the ligand for Ni-MOFs grown on carbon cloth. The presence of the amino group in HATP·6HCl facilitated the catalytic oxidation of glucose and its interaction with other ligands, thereby enhancing the electrochemical response of Ni-MOF to glucose. The material exhibited a pore size predominantly ranging from 2 to 9 nm, with a specific surface area of 207.15 m^2^g^−1^. During the CV curve testing, the peak current was found to be the highest at pH 14. However, excessively high alkalinity could potentially damage the electrode, so a solution with pH 13 was selected for subsequent tests. The optimal working potential of the electrode was determined to be 0.6 V, which was higher than the commonly used 0.55 V. The material demonstrated a linear relationship with high concentrations of glucose, and the detection limit was measured to be 0.57 μM (S/N = 3). To evaluate the material’s resistance to interference, common biomolecules and Cl^−^ were selected for testing, and it was found that their addition had minimal impact on the material’s current response. However, it should be noted that the material’s current response to human serum has not been compared against clinical reports or standard commercial glucose sensors. Cobalt–nickel nanowires were grown on carbon cloth using one-step hydrothermal method, and ZIF-67 structures were grown in situ on the nanowires after annealing. Tao et al. [80] showed that the crystal structure of ZIF-67, upon the addition of Go (graphene oxide), becomes irregular, and the crystal size begins to decrease. This may be due to the reaction of the oxygen-containing group of Go with ZIF-67. The XPS peak of the material shows that the peak at 531.23 eV indicates oxygen vacancy, which was considered to be related to glucose response. The detection limit was 0.16 μM, the sensitivity was 990.12 μA mM^−1^ cm^−2^, and the linear response range was 0.3 μM–5407 mM. The anti-interference line test did not include common Cl^−^ ions, which may reduce the persuasiveness of the study’s findings. However, the material’s excellent stability was demonstrated over a period of 30 days, confirming its long-term stability.

**Figure 4:**
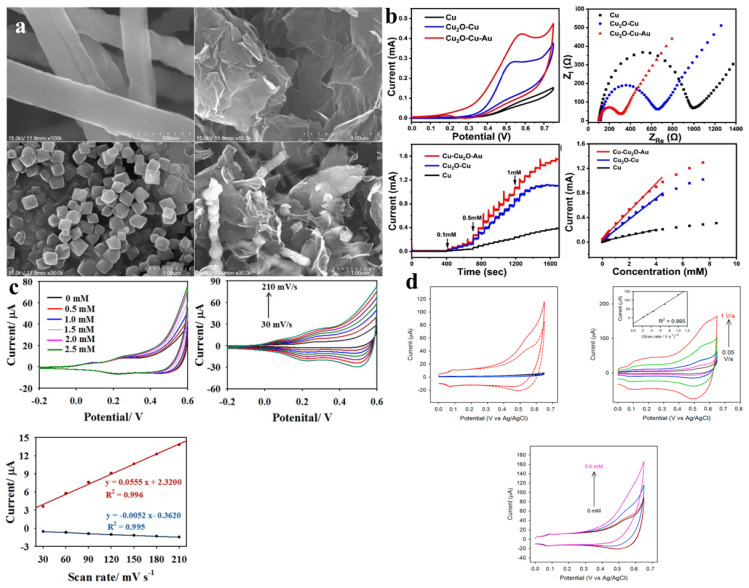
(**a**)The SEM images of Cu NWs, GO, MOFs, and Cu NWs−MOFs−GO hybrid nanocomposite [81]. (**b**) CV curves and the electrochemical impedance spectra of Cu/Nafion/GCE, CCu/Nafion/GCE, CCAu/Nafion/GCE electrode [82]. (**c**) CV curves of N−Co−MOF@PDA−Ag modified electrode and corresponding plots of redox peak currents vs. scan rates [83]. (**d**) CV curves with different conditions [84].

A new CuNW(nanowire)-Go-MOF composite was used to test the electrochemical response to glucose [81]. SEM images are shown in Figure 4a. The material was obtained via the sonication of Cu NW, Go, and MOF in solution. A bare gold electrode was chosen as the control electrode for this material. The composite had a low detection limit of 7 μM and a linear response range of 20–26.6 μM. Its excellent performance may be due to the large specific surface area of the octahedral porous MOF and the good conductive conditions provided by Go. Subsequent stability and selectivity experiments conducted on the material demonstrated its ability to maintain stable responses to glucose across various electrodes. When uric acid, acetaminophen, and ascorbic acid were used as interfering substances at much higher concentrations than normal human blood, the sensor showed a high response only to glucose. When the material was subjected to Cl^−^ poisoning experiments, the sensor’s response to glucose was not affected by the high Cl^−^ concentration. The CV curves and the electrochemical impedance spectra of Pu’s word are shown in Figure 4b. The main performance parameters of a glucose sensor based on MOF composite are shown in Table 3.

**Figure 5:**
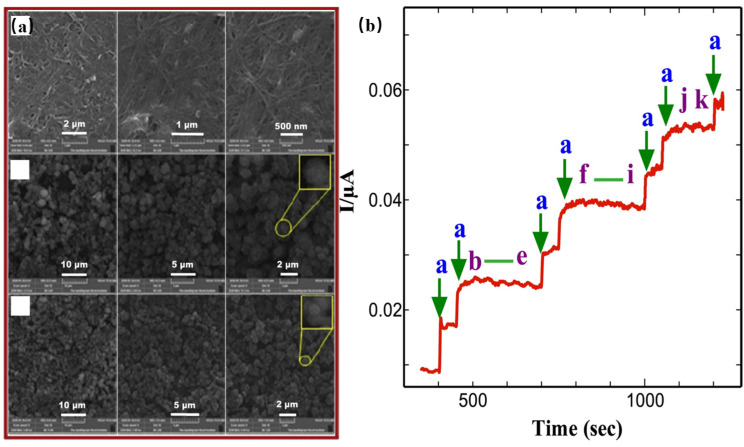
(**a**) SEM images of Arul’s materials; (**b**) amperometric i–t curve responses for adding 2 μM glucose (a); adding 200 μM each AA, UA, DA, and urea (b–e); adding 800 μM each Na^+^, Cu^2+^, Mg^2+^, Cl^−^, NO^3−^, and SO_4_^2−^ (f–k). [93].

The researchers generated Ni-Co bimetallic MOFs on carbon cloth [56]. The MOF had a mesoporous structure with a specific surface area of 180.3 m^2^/g and a pore volume of 0.39 cm^3^/g. The XPS spectra showed that Ni and Co existed only in a divalent state, and the material had no Ni^3+^ and Co^3+^. The CV curves were measured to evaluate the materials. It was observed that when the Ni-Co ratio was less than 1:1, the oxidation potential increased, and the current density for glucose was higher. Conversely, when the Ni-Co ratio exceeded 1:1, the opposite trend was observed. This behavior could be attributed to the higher conductivity of Ni compared to Co, and the addition of Co to Ni reduced the electronic transition difficulty in the material. Notably, the material with a Ni:Co ratio of 2:1 exhibited the highest current density. The structural characteristics of MOFs contribute to their high specific surface areas, enabling the provision of more active sites for glucose adsorption. The CV curves obtained at different scan rates demonstrated that the position of the anodic peak shifted with an increasing scan rate, indicating that the glucose diffusion rate on the electrode surface was kinetically limited. The material exhibited an optimum working potential of 0.5 V, and the electrode could respond to changes in glucose concentration within 2 s. Furthermore, the material’s anti-interference capability, stability, and performance in detecting actual samples were investigated. The test of the actual samples was not compared with the clinical report. The CV curves of Zhai et al. [83] and Mo et al. [84] are shown in Figure 4c,d. Guo et al. [92] prepared Co_3_O_4_/NiCo_2_O_4_/CC. The nanowires provided by NiCo_2_O_4_ allow for electron transfer between CC and Co_3_O_4_. The specific surface area of the material was extensive, and the large specific surface area provided abundant active sites. The optimum working potential of the material was 0.55 V in 0.1M NaOH solution. The linear response to glucose was in the range of 0.001–1127 mm. The detection limit was 0.64 μM (S/N) = 3. The material also had good selectivity and stability. Arul et al. [93] prepared SWCNTs-MPsLCu-MOF on the electrode via two-step electrodeposition. Firstly, SWCNTs/GCE was prepared by electrodepositing GCE into solution and then putting the prepared SWCNTs/GCE into a copper nitrite solution for electrodeposition to prepare the final product SWCNTs-Cu-MOF/GCE. In order to understand the effect of SWCNT on morphology, the researchers also directly prepared Cu-MOF/GCE by one-step electrodeposition, Figure 5a. The results showed that SWCNTs did not affect the morphology of Cu-MOF. The experimenters investigated the impact of various deposition times on the glucose oxidation peak and observed the following findings: when the deposition time was less than five minutes, both the oxidation and reduction peaks were achieved within this timeframe. However, when the deposition time exceeded five minutes, the oxidation current began to decrease once again. The former may be caused by the short deposition time, lower surface Cu-MOF, and insufficient active sites. The observed reduction in oxidation current when the deposition time exceeds five minutes may be attributed to the prolonged duration of deposition. This extended deposition time could lead to an uneven distribution of the Cu-MOF coating on the electrode surface, resulting in a reduction in the surface-active sites. To detect the selective response of the material to glucose, firstly, an alkaline solution containing 200 μM ascorbic acid, uric acid, dopamine, and other impurities was prepared, and 2 μM glucose was added in batches. The current intensity only increased when glucose was added, indicating that the material had a strong selectivity to glucose (Figure 5b). Real samples were tested using human saliva, and the results differed very little from the clinical testing device.

Cao et al. [94] used a reduction method to dope Ag into Ni-MOF, which was hydrothermally prepared. The Ni-MOF was a three-dimensional layered nano-flower, and the morphology of the Ag-doped MOF did not change essentially. An EDX analysis of the material gave an approximate Ag doping of 6.9%. After performing the BET analysis, it was determined that the Ag-doped MOF displayed a greater specific surface area and pore size in comparison to the Ni-MOF. A large specific surface area was favorable for glucose reaction, and a large pore size was favorable for electron transfer and ion diffusion. The Nyquist plot of Ag@Ni-MOF exhibited smaller semicircles and steeper straight lines at lower frequencies. The results showed that Ag@Ni-MOF has a low resistance, and thus the diffusion limiting process was accelerated. The Ag@Ni-MOF has a linear response to 5–500 μΜ glucose. The electrode’s sensitivity was 168.08 μA mM^−1^ cm^−2^, and the LOD was 5 μM (S/N = 3). The selective response of the electrode to glucose was studied using some common interfering agents, and the results showed that the influence of these substances was negligible. In addition, the material can also be used as a supercapacitor. Other researchers added Au particles to Ni-MOF [85]. The solution was dropped into the prepared MOF glycol solution under ultrasound, and the mixed solution was heated in a microwave oven for 30 s, washed, and dried to obtain the product Au@NiBTC. The surface roughness and specific surface area of the material increased after adding Au. The material performed better than the material with added Ag in the Ni-MOF. For instance, the detection range of the material was found to be 5–7400 μM, with a sensitivity of 1447.1 μA mM^−1^ cm^−2^ and a minimum detection limit of 1.5 μM (S/N = 3). While Cu_2_O exhibits high stability and inherent properties, its catalytic oxidation performance for glucose is generally modest. However, the formation of a core–shell structure by combining Co with ZIF-67 through the ultrasonic method can enhance Co’s oxidation ability towards glucose [95]. The addition of Cu_2_O does not alter the morphology of ZIF-67, and Cu_2_O is encapsulated within the MOF structure. The presence of cuprous oxide contributes to an enhanced response current, potentially due to the oxidation of Cu from Cu^+^ to Cu^2+^ and Cu^3+^. The CV curve of the material after the addition of Cu_2_O also displays an oxidation peak at 0.35V, indicating an improved oxidation ability resulting from the inclusion of Cu_2_O. Among the synthesized materials, Cu_2_O@ZIF-67 exhibits the highest sensitivity (307.02 μA mM^−1^ cm^−2^) and the lowest detection limit (6.5 μM). Even though Cl^-^ was not included in the anti-interference test of the material, it exhibited excellent stability.

### 3.2. Enzyme-Free Glucose Sensor Based on MOF Derivatives

The researchers additionally explored the design of MOF derivatives by integrating them with foams featuring mesoporous channels or incorporating biomolecules such as porphyrins. Furthermore, functionalization techniques were employed to enhance the electrocatalytic performance of MOFs for glucose detection. These MOF-derived materials offer several advantages over the original MOF materials, including improved conductivity, enhanced stability, and a uniform distribution of active centers. An innovative wet-spinning method was employed to fabricate a flexible fiber bimetallic MOF electrode, which can be directly affixed to the human arm for the detection of sweat glucose concentration [96]. The fiber electrode has good ductility. The CV curve applied to the material under static strain shows that the oxidation peak current was reduced to 80% under 100% tension. The CV curve of the material does not change significantly after up to 1000 deformations. The experimenters tested the effects of different MOF coating amounts, rGO and PU mass ratios, fiber length, and potentials on the current glucose response. The results showed that the optimal MOF coating amount was 4.5 mg/mL, the optimal rGO and PU mass ratio was 1:5, the optimal fiber length was 5cm, and the optimal test potential was 0.5 V. The sensitivity of the material was 425.9 μA mM^−1^ cm^−2^. The detection limit was 3.28 μM. DA, LA, NaCl, and UA were selected for the anti-interference test of materials. The addition of these substances had little effect on the current. The material can rarely produce a good electrochemical response in a neutral solution. The researchers recruited volunteers to participate in the wearable sweat glucose monitoring experiment, which provided an intuitive assessment of the sweat glucose concentration of the volunteers both before and after meals. The results were very close to those of commercial blood glucose meters.

Distinguishing itself from previous studies, the study of Ding et al. involved MOFs (CoO, Fe_3_O_4_, NiO, CuOx, and ZnO) that were prepared on copper foams and then calcined in a tube furnace to obtain new products, CuXON/CF [60]. The TEM images showed that the products had a core–shell structure. The Co element was mainly in the core, and the Cu element was in the shell. The specific surface area of calcined CuXON/CF (27.17 m^2^g^−1^) was much smaller than that of uncalcined CuXON/CF (736.78 m^2^g^−1^). The total pore volume decreased after calcination from 0.2888 cm^2^g^−1^ to 0.0959 cm^2^g^−1^. Compared with CuXON/CF, the electrochemical performance of the copper foam without MOF was improved greatly. It may be that Cu^2+^ provides extra electrons to Co_3_O_4_ in the core. The core–shell structure can also bring more catalytic active sites and improve the electrochemical sensing performance of the material. The Nyquist plots of CuCoON/CF showed that the ECSA of CuCoON/CF was larger than that of other materials, which should be due to the increase in the shell provided by CuOx for Co. The optimal potential of CuCoON/CF for glucose sensing was 0.25 V, which was half of that of other materials. The sensitivity was 27,778 μA mM^−1^ cm^−2^. The concentration range tested for CuCoON/CF on glucose was 0.1–1300.0 μM. The detection accuracy of the material for human serum was also verified. Layered double hydroxides have the characteristics of a large specific surface area and high redox activity. Combining the material and the MOF can improve the electrocatalytic performance of the MOF [88]. The Au@NiCo LDH was synthesized using a hydrothermal method by incorporating Au into the prepared NiCo-MOF. Subsequently, the oxidation peak of the material exhibited a significant increase upon the addition of Au. Notably, the sensitivity of this material (864.7 μA mM^−1^ cm^−2^) surpassed that reported in Shu’s work [96], and the lowest detection limit was also lower (0.028 μM (S/N = 3)). The anti-interference and stability of the material were also very good.

**Figure 6:**
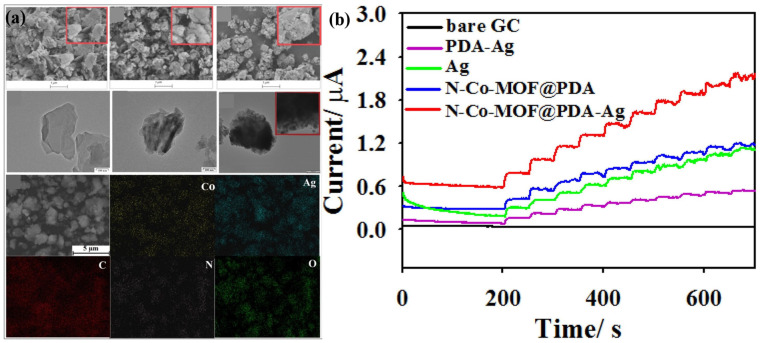
(**a**) SEM images and the corresponding mapping images of N-Co-MOF, etc. (**b**) The amperometry responses with successive addition of 6 μM glucose [83].

It is worth mentioning that Zhai et al. [83] synthesized N-Co-MOF@PDA-Ag, N-Co-MOF@PDA, and PDA-Ag. N-Co-MOF was prepared via a hydrothermal reaction, and then the dried product was dispersed in Tris buffer solution (pH 8.5, 10 mM), and PDA was added to synthesize N-Co-MOF@PDA. Finally, the product was poured into a silver ammonia solution and stirred in the dark to obtain N-Co-MOF@PDA-Ag. In contrast, the preparation of PDA-Ag was much simpler. PDA was added to the buffer solution, followed by dropping silver ammonia solution in the dark to obtain PDA-Ag. The three products’ SEM, TEM, and EDS images are shown in Figure 6a. The CV curve was experimentally tested in the absence of a glucose solution, revealing the presence of a redox peak corresponding to the conversion between Co^2+^ and Co^3+^ ions. In contrast, the bare electrode did not exhibit any redox peak. Subsequently, the current response to glucose was measured at 0.55 V for different electrodes, and the results are presented in the figure. Among them, N-Co-MOF@PDA-Ag demonstrated an excellent response to glucose. Furthermore, the effect of varying Ag+ ion concentrations on the material’s properties was investigated. It was determined that a concentration of 5.0 mg/mL was optimal. The amperometry responses are shown in Figure 6b. The authors offered an explanation stating that below this concentration, the low composite content does not significantly contribute to the current response, while above this value, Ag nanoparticles may be present outside the N-Co-MOF@PDA-Ag structure. The selective response of the material to glucose and the response to human serum were also tested. The concentration response of real samples was consistent with the hospital test report, proving the feasibility of its practical application. The main performance parameters of a glucose sensor based on MOF derivatives are shown in Table 4.

**Figure 7:**
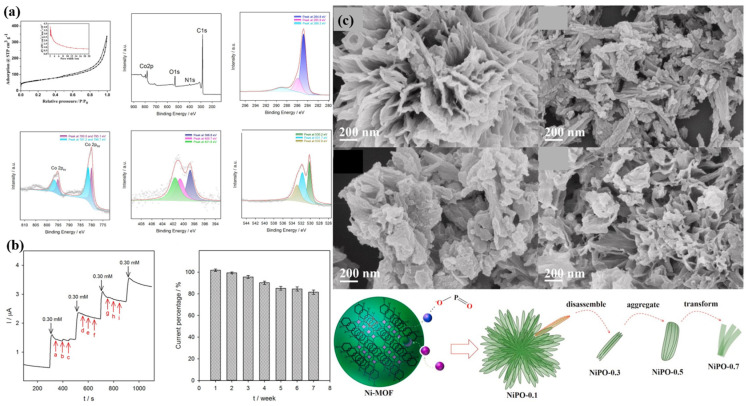
(**a**) N_2_ adsorption-desorption isotherm of NHCN-Co_3_O_4_, and XPS spectrum of NHCN-Co_3_O_4_ [117]. (**b**) Selectivity test at 0.45 V (a, b, c, d, e, f, g, h, and i: 3.0 μM UA, AA, DA, 0.30 mM leucine, glutamic acid, 0.30 mM alanine, pyruvic acid, lactic acid, and KCl); stability testing [117]; (**c**) SEM images of NiPO [114].

Yan et al. [118] first prepared ZIF-67, which was then calcined to obtain the product hollow dodecahedron Co_3_O_4_. PDA was then polymerized on dodecahedron Co_3_O_4_. The experiment was also performed by directly coating PDA onto ZIF-67 to obtain the product. Electrochemical impedance spectroscopy was used to compare the electron transfer capabilities of the two products (ZIF-67/ITO and Co_3_O_4_/ITO). The results showed that the resistance of Co/CoO/N-C was the smallest, which may be due to the more catalytic sites and better conductivity being provided by PDA. The best working potential was 0.55 V, and the best concentration of NaOH was 0.6 M. The high concentration of OH^-^ resulting from the higher pH may hinder the adsorption of glucose on the electrode. Moreover, high concentrations of sodium hydroxide increase the background current. During testing using the amperometry method, it was observed that as the glucose concentration increased to a certain extent, the response exhibited a slow rate of increase. This phenomenon could potentially be attributed to the occupation of active sites by some glucose molecules. The experimenters tested the anti-interference ability, stability, and response to actual materials. The tests on human serum were not compared with clinical reports. As above, Ouyang et al. [117] also prepared a non-enzymatic glucose sensor using ZIF-67. The difference was that Ouyang et al. used a two-step redox pyrolysis to dope N into Co_3_O_4_ hollow nanoparticles. The XPS spectra showed that the principal Co oxide was Co_3_O_4_, and the proportion of Co^2+^ was higher than that of Co^3+^, Figure 7a. An analysis of the XPS peak of the oxygen element showed that the peak at 532.9 eV was relatively large, and the peak at this position was related to the oxygen vacancy. This feature may also enhance the catalytic ability of the material. When performing electrochemical tests, it was found that the best current response was at 0.45 V, which was better than in previous reports. The material can generate a current step for 1 μM glucose within 3 s at a potential of 0.35 V, and the maximum detection limit can be as high as 32 mM. Materials can detect high glucose concentrations to provide new methods for detecting clinical diabetic patients. After calculation, 0.2 μM was the minimum detection limit of the material. The assessment of the anti-interference ability primarily focuses on chloride ions and various typical biological macromolecules, such as amino acids. These selected substances have been shown not to interfere with the accurate detection of glucose, as depicted in Figure 7b. The experimenter conducted six human serum tests; the results were similar to the clinical report. The stability of the material was tested within three weeks, and the attenuation of the current density response was less than five percent within three weeks.

Mo’s [84] work pyrolyzed ZIF-67 in a reducing atmosphere to obtain Co nanoparticles (Co@NCD). The electrode fabrication was relatively complicated, and the polished GCE was sonicated in pure water and acetone, and then a 0.05% Nafion solution of Co@NCD was dropped onto the GCE. The electrode prepared with this material had a minimum oxidation current of 0.2 V, much lower than the 0.35 V above. Increasing the glucose concentration at a 0.2 V potential also established an excellent linear correlation. At the optimal potential of 0.5 V, the response can be generated within 1 s. The minimum detection limit of the material was 0.11 μM, which is lower than the 1 μM mentioned above, and it was also a derivative of ZIF-67. Similarly, the experimenters tested the anti-interference properties of the material, and the effects of uric acid (UA), ascorbic acid (AA), and dopamine (DA) on the current density were negligible. Notably, the interference of Cl^−^ on the material was less than 3%. The practicality of the method was proven by the fact that the error between the detection of human serum and the corresponding clinical report was less than 3%. Flower-like, rod-like, and ribbon-like nickel phosphates were prepared based on spherical Ni-MOF [114], Figure 7c. Firstly, Ni-MOF was prepared using the hydrothermal method. Subsequently, the prepared Ni-MOF was combined with NaH_2_PO_2_·6H_2_O and different concentrations of Ni(NO_3_)_2_·6H_2_O for a secondary hydrothermal treatment, resulting in the formation of various morphologies of NiPO. The XPS spectra revealed that the nickel in the obtained material exhibited a rod-like shape and contained a higher valence state. Interestingly, the rod-like nickel phosphate exhibited a sensitivity to 0.1 mM glucose that was 1.8 times higher than that of Ni-MOF. On the other hand, the flower-shaped and ribbon-shaped nickel phosphates did not demonstrate a significant response to 0.1 mM glucose. Similar to other derivatives of ZIF-67, the lowest detection limit for the material was calculated to be 1 μM, with a sensitivity of 3169 μA mM^−1^ cm^−2^. Notably, the rod-shaped nickel phosphate achieved its highest sensitivity at pH 13, distinguishing it from other forms of the material. The experiments assessed the material’s ability to resist interference. However, Cl^−^ were not used as interfering molecules. Additionally, only some biomacromolecules were tested, such as sugar, lactose, sucrose, ascorbic acid, dopamine, and uric acid. Even so, the concentration of the interferent chosen by the experimenter, 0.01 mM, was one-tenth that of glucose, 0.1 mM. No comparison with clinical reports has been made in human serum tests. In conclusion, it can be concluded that the selectivity of this material towards glucose and its practical application were not optimal.

**Figure 8:**
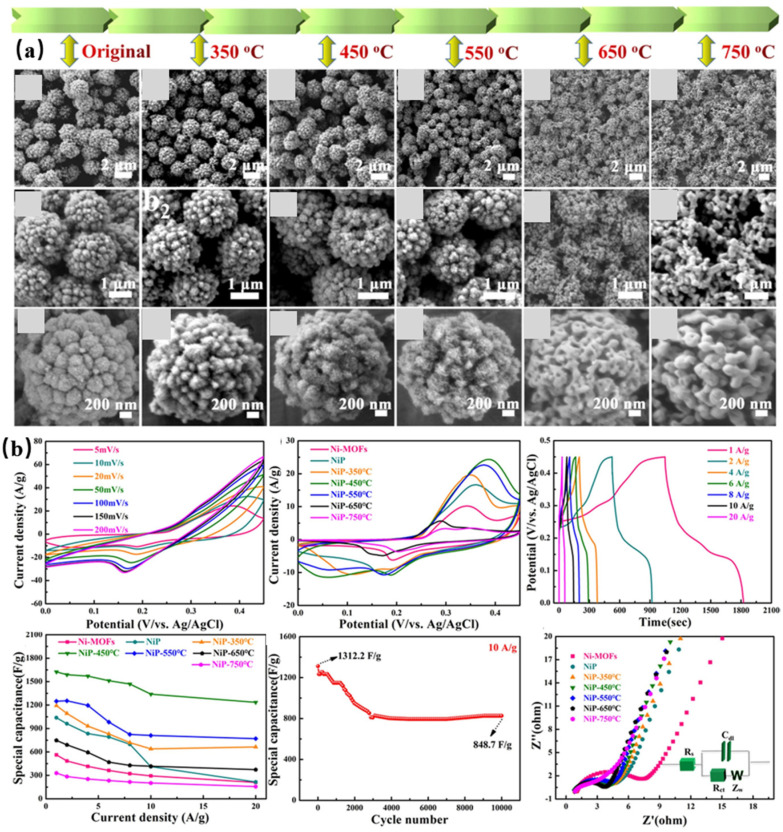
(**a**) FESEM images at different magnification of Ni−MOF and NiP [113]. (**b**) CV curves and Nyquist plots [113].

Ma et al. [113] employed a hydrothermal method to synthesize Ni-MOF, which was subsequently used for secondary hydrothermal preparation of NiP by immersing the Ni-MOF in a solution. The resulting product was subjected to calcination in an inert gas atmosphere at temperatures of 350, 450, 550, 650, and 750 °C to obtain the final product. Figure 8a presents the scanning electron microscopy (SEM) image of the phosphorylated MOF. From left to right in the image, an increase in temperature is observed, leading to a greater blooming degree of the flower-like structure. Notably, the flower structure reaches its maximum size at 450 °C. With a further temperature increase, the crystal aggregation becomes more pronounced, resulting in the formation of larger aggregates or lumps. The pore size distribution of the tested material shows that the maximum specific surface area of the product annealed at 450 °C was 148.1m^2^g^−1^. The thermogravimetric analysis of the material at 25–790 °C showed that the maximum weight loss occurred at 25–350 °C, which should be due to the removal of water molecules in the material. The 4% weight loss at 400–550 °C was due to the decomposition of organic residues. There was no vacancy peak in NiP-450 at O1s. During the CV test, it was observed that all seven materials exhibited a distinct redox peak in the solution, attributed to the conversion between Ni2+ and Ni3+ ions. Notably, NiP-450 demonstrated the highest current response in the glucose solution due to its large specific surface area and small particle size. To further investigate the performance of the electrodes, the researchers conducted an electrochemical experiment using different concentrations of NiP-450 under identical conditions. Through their analysis, they determined that an optimal electrode modification level of 2.0 mg/mL was crucial, as exceeding this amount could potentially impede glucose contact with the electrode. Consequently, to achieve optimal performance, a voltage potential of 0.55 V was identified as the most suitable choice. Further testing found that 0.1 M NaOH could increase the OH^-^ concentration required for optimal electrochemical characterization. The lowest detection limit of the material was 0.16 μM. CV curves and Nyquist plots shown in Figure 8b. However, it should be noted that the detection range of this material was relatively narrow, and the current response slowed down below 5.1 mM. These limitations may pose challenges in practical applications, particularly for diabetic patients who may experience low blood glucose levels. The stability of the material has also proven excellent. The immunity test also did not introduce Cl^−^, which makes the data unconvincing. The excellent catalytic performance of this material was attributed to (1) the high specific surface area and shell permeability improving the electron transfer efficiency; (2) the laminated structure of the nanosheets preventing agglomeration and providing more reaction checkpoints; and (3) the electron transfer resistance being reduced following annealing.

## 4. Conclusions and Outlook

This review is focused on non-enzymatic glucose electrochemical sensors that utilize MOFs and their derivatives. MOF materials possess a high specific surface area attributable to their mesoporous structure. This unique structure provides adsorption sites for glucose within both the inner and outer pores, enabling glucose catalysis when subjected to an electric current, thus resulting in an electrochemical response. Researchers have explored the synthesis of core–shell structures of MOFs to further enhance their conductivity. Notably, investigations have included the combination of two MOFs to form core–shell structures [58,63], the integration of MOFs and carbon materials into core–shell architectures [73], and the fabrication of core–shell structures incorporating noble metal Ag and MOFs [94]. These engineered structures significantly enhance both the material’s conductivity and active sites, thereby fundamentally improving the catalytic performance of glucose. Other researchers explored additional strategies. For instance, varying the sonication time [40] has been employed to effectively increase the specific surface area of MOFs, thereby amplifying the number of active sites available for glucose interaction. Moreover, researchers have investigated the combination of glucose with porphyrins, which offers a synergistic effect to improve sensor performance. Another approach involves the integration of glucose with diverse carbon-based materials, presenting a promising avenue for performance enhancement. For example, the synthesis of glucose on carbon substrates and the incorporation of MOFs with different forms of carbon materials have been explored, demonstrating the potential to achieve notable improvements in glucose sensing capabilities. The preparation of various hierarchical structures of MOFs to increase the specific surface area and thus improve the performance of glucose is also one of the methods.

Thus far, the primary endeavor of researchers has been directed towards enhancing the accuracy and sensitivity of glucose detection techniques, with a specific emphasis on expanding their detection range, facilitating a rapid response, and ensuring selectivity. However, the ultimate objective also encompasses the advancement of flexible sensors that can unlock new possibilities for monitoring athlete sweat. Noteworthy among these pioneering approaches is the work of Xuan et al. [40], who have successfully developed a flexible non-enzymatic glucose sensor based on MOFs and attempted to employ it for monitoring sweat levels.

## Figures and Tables

**Table 1:** Enzyme-free Glucose Biosensor Based on Monometallic MOF.

Sensor Materials	Real Samples	Linear Range (mM)	LOD (μM)	Sensitivity (μA mM^−1^ cm^−2^)	Ref
Ni-MOF	-	-	0.66	21,744	[39]
Ni-MOF	sweat glucose	0.001–0.4	0.89	3297.1	[40]
Cu-MOF	urine	0.06–5000	0.01	-	[41]
CuO nanowires	-	-	0.002	648.2	[42]
Cu-MOF	human serum	0.001–0.95	0.076	30,030	[43]
Ni-MOF		0.001–1.6	0.46	2859.95	[44]
Co-MOF	-	-	0.3	-	[45]
Co-MOFs	-	10–1200	3.2	160.75	[46]
Co-MOF	-	0.005–0.9	1.6	169	[47]
Ni-MOF	human serum	0.025–3.15	0.6	402.3	[48]
CuO architectures		2.8	0.1	934.2	[49]
CuO	human serum	0.0005–5.0	-	-	[50]
Cu-MOF/CPE	human serum	0.005–10.95	0.11	-	[51]
Co-MOF/EG	-	1–3330	0.58	23,330	[52]

**Table 2:** Enzyme-free Glucose Biosensor Based on Multi-Metal MOF Combination.

Sensor Materials	Real Samples	Linear Range (mM)	LOD (μM)	Sensitivity (μA mM^−1^ cm^−2^)	Ref
Ni/Co-MOF	human serum	0.3–2.312	0.1	3250	[56]
Ni/Co-MOF	human serum	0.1–1400	0.047	2086.7	[57]
Ni@Cu-MOF	human serum	0.005–2.5	1.67	1703.33	[58]
Cu-Co ZIF derivative	human serum	0.02–0.8	-	18,680	[59]
CuOx@Co_3_O_4_	human serum	0.0001–1.3	-	27,778	[60]
UiO-67@Ni-MOF	human serum	5–3900	0.98	-	[61]
NiCu-MOF-6	human serum	0.02–4.93	15	1832	[62]
Cu@ Ni MOF	human blood serum	0–5	0.0004	496	[63]
Co_3_(BTC)_2_ MOFs		1–330	0.33	1792	[64]
ZIF67/ZIF8	-	-	6.5	833.61	[65]
Cu/Co-ZIF-20	-	0.05–6	-	0.03	[66]
ZIF-Zn0.5Co0.5		up to 1.25	9	1105.6	[67]
Cu_1_Co_2_-MOF/Ni foam		0.05–0.5		8304.4	[54]

**Table 3:** Enzyme-free glucose electrochemical sensor based on MOF composite material.

Sensor Materials	Real Samples	Linear Range (mM)	LOD (μM)	Sensitivity (μA mM^−1^ cm^−2^)	Ref
CuO polyhedrons/CC	-	0.5–800 μM	0.46	13,575	[36]
Co–Ni–C-MOF	-	5–1000	0.75	1964	[62]
Bimetallic NCNT MOF CoCu nanostructure	-	0.05–5.5	0.00015	1027	[68]
NiCo_2_O_4_ HNCs	human serum	0.00018–5.1	0.027	1306	[71]
Ni/NiO@C	blood serum	0.01–2	0.116	1291	[74]
NiO/C-MOF	-	0.005–4.1	0.92	2918	[78]
Ni-MOF NSAs/CC	human blood serum	0.001–7	0.57	13,428.89	[79]
Cu NWs-MOFs-GO	human serum	26.6	0.007	-	[81]
Cu_2_O-Cu-Au	human serum	-	1.71	1 × 10^6^	[82]
N-Co-MOF@ PDA-Ag	human serum	0.001–2	0.5	183.6	[83]
Co@NCD	human serum	0.0002–12.0	0.11	125	[84]
Au@Ni-MOF	bovine serum	0.0005–1	0.36	246.8	[85]
Ag@In_2_O_3_/NF	-	10 μM–2.16 mM	0.49	3310	[86]
ZnCo_2_O_4_ microrice	-	0.55–2.65	5	215.1	[87]
Au@NiCo LDH	-	0.005–12	0.028	864.7	[88]
CoZn-LDHs	-	0.001–0.255	15.6	1218	[89]
Cu/g-SiCNT/CuO	-	0.001–4.48	0.8	2051	[90]
ZIF-67/rGO/CF	-	0.0001–1	0.2	-	[91]

**Table 4:** Enzyme-free glucose sensor based on MOF derivatives.

Sensor Materials	Real Samples	Linear Range(mM)	LOD (μM)	Sensitivity (μA mM^−1^ cm^−2^)	Ref
Ni_3_(HITP)_2_ MOFs	-	0–10	-	-	[37]
3D M-BDC-MOF	-	0.01–0.8	6.68	636	[38]
NiCo LDH/NiCoS/CC	-	0.001–3, 4–9	0.208	2167, 1417	[69]
Ni/Co-TCPP-MOF	human serum	0.001–3.8	0.3	2800	[70]
NiO/Cu-TCPP	-	0.00285-0.2885		4666	[72]
Ni_3_N@C	bovine serum	1–3000	0.3	1511.59	[73]
Ni/Ni(OH)_2_-NFs/CP	-	0.2–60	800	1078	[74]
MOF-74(Cu) NS-CC	-	0.1–1	0.14	381	[75]
Ni@NC7H	human serum	0.001–1.805	0.34	1440	[76]
Ni/NiO/NG-400	Human serum	0.001–3.568	0.032	3251.8	[77]
ZIF-67@GO/NiCo_2_O_4_/CC	-	0.0003–5.407	0.16	990.12	[80]
Hierarchical Co_3_O_4_/NiCo_2_O_4_/CC	human serum	0.001–1.127	0.64	12,835	[92]
SWCNTs-MPsLCu-MOF	human saliva	0.000020–0.08	1.72	573	[93]
Ni–Co MOF/Ag/rGO/PU	sweat glucose	10–660	-	425.9	[96]
CoFe-PBA/Co-ZIF/NF	-	1.4–1500	0.02	5270	[97]
FeBDC-derived Fe_3_O_4_	-	Up to 9.0 mM	15.7	4670	[98]
CuO-350-NA/GCE	-	5–1165	0.63	1614.4	[99]
NiCo_2_O_4_ nanowire arrays/Ni foam	human serum	0.001−3.987	-	5916	[100]
Co–Ni–C-MOF	-	5–1000	0.75	1964	[101]
Cu@HHNs	-	5–3000	1.97	1594.2	[102]
SPCE\15 %CuO−IL	-	1–2800	1	820	[103]
CC@MOF-74(NiO)@NiCo LDH	-	10–1100		1699	[104]
α-CD-rGO/Ni-MOF/TM	-	0.65–4.828	0.3	1395	[105]
Nafion/Co/MnO@HC/GCE	-	Up to 6.9	1.31	233.8	[106]
Ag@TiO2@ZIF-67	-	-	0.99	7880	[107]
Ti_3_C_2_Tx/ZIF-67	-	0.005–7.5	3.81	-	[108]
ZIF-67 HNPs	-	0.005–3.3	0.96	445.7	[109]
HS-ZIF-67	-	-	3.9 × 10^–6^	1074.22	[110]
Ag@ZIF-67@MWCNT/NF		0.033–0.44	0.49	13,014	[111]
Ni/Ni(OH)_2_-NFs/CP	-	0.2–60	800	1078	[112]
NiP-450	-	0.001–0.6	0.16	1373.7	[113]
r-NiPO	human serum	0.001–3	1	3169	[114]
NiO-NC@rGO/GCE	-	0.5–20	0.07	4254	[115]
Ni_3_S_2_@NCNT	-	0.46–3190	0.14	1447.64	[116]
NHCN–Co_3_O_4_	human serum	0.001–32.0	0.2	12,900	[117]
Co/CoO/NC	-	0.01–2.75	0.8	143.9	[118]
NN-CuO/N-rGO	-	0.5–639	0.001	3400	[119]
Porous copper@carbon agglomerate	-	0.005–3.33	0.29	614.3	[120]
Integrated Co(OH)_2_/GCE	human serum	0.005–6.7	1.73		[121]

## Data Availability

Not applicable.
